# A Feasibility Study of Wearable Activity Monitors for Pre-Adolescent School-Age Children

**DOI:** 10.5888/pcd11.130262

**Published:** 2014-05-22

**Authors:** Sara E. Schaefer, Marta Van Loan, J. Bruce German

**Affiliations:** Author Affiliations: Marta Van Loan, Western Human Nutrition Research Center, University of California, Davis; J. Bruce German, Foods for Health Institute, University of California, Davis, Davis, California.

## Abstract

**Introduction:**

Understanding physical activity is key in the fight against childhood obesity. The objective of this study was to examine the feasibility of using certain wearable devices to measure physical activity among children.

**Methods:**

A qualitative study was conducted with 25 children aged 7 to 10 years to assess acceptability and compliance of wearable activity devices in this age group. During March through August 2012, children participated in a 4-week study of 3 accelerometer models and a heart rate monitor. Children were asked to use a different device each week for 7 consecutive days. Children and their parents completed structured interviews after using each device; they also completed a final exit interview.

**Results:**

The wrist-worn Polar Active was the device most preferred by children and was associated with the highest level of compliance. Devices that are comfortable to wear, fit properly, have engaging features, and are waterproof increase feasibility and are associated with higher levels of compliance.

**Conclusion:**

The wrist-worn device was the most feasible for measuring physical activity among children aged 7 to 10 years. These findings will inform researchers in selecting tools for measuring children’s physical activity.

## Introduction

Effective health interventions in youth contribute to the lifelong prevention of obesity and related metabolic diseases (1–4). In general, regular physical activity improves the physiologic, metabolic, and immunologic processes, as well as quality of life of individuals (5). Accurate measurement of physical activity is useful in evaluating health risks in populations of interest and facilitating appropriate interventions (6). Objective methods for measuring physical activity are preferred over subjective methods because of variations in perceptions and reporting among individuals (7). Wearable devices, such as pedometers that measure physical activity quantity, are practical in that they are low cost and relatively small in size. But physical activity quality is also an important variable in health (8). When worn properly, accelerometers provide objective data on both the quantity and quality of physical activity. For this reason, accelerometers are increasingly used in health research to provide more complete, accurate, and objective information on physical activity patterns of free-living study populations (9,10).

Accelerometers are becoming increasingly available commercially and emerging with various specifications, styles, and attachment sites. The research context and characteristics of the target population are important considerations in selecting appropriate wearable tracking devices (11–13). Young populations, for example, warrant different sets of compliance factors than adult populations (14–16). 

Little information is available on the acceptability of accelerometer use in pre-adolescent youth. This study evaluates the relative ease of using accelerometers in this population, including examination of device acceptability, adherence to protocol, frequency of lost or missing data, and barriers to their use (cost, skill level, reactivity, etc.) that may affect feasibility decisions. (17,18). We also examine various device data feeds and formats as appropriate for youth research and engagement purposes for health and fitness.

## Methods

This study was conducted in Yolo County, California, during March through August 2012 and was approved by the institutional review board of the University of California, Davis (UC Davis).

Several commercial devices were selected to provide a range of specifications, styles, and attachment sites. Two accelerometer types were provided by the US Department of Agriculture’s Western Human Nutrition Research Center (WHNRC) physiology laboratory: the Philips Actical (Philips North America Corporation, Andover, Massachusetts) and the SenseWear Pro2 (BodyMedia, Inc, Pittsburgh, Pennsylvania). Several units of the Polar Active (Polar Electro Inc, Lake Success, New York) were donated by Polar Electro to the Foods for Health Institute at UC Davis. A heart rate monitor by Polar (Polar Electro Inc, Lake Success, New York) was also provided by the WHNRC for assessment because information on its use in children may guide future child health studies.

We assessed the costs and skills required to use each device and the features that may contribute to feasibility decisions (Table). Both the Philips Actical and Polar Active models contain uniaxial accelerometers with built-in algorithms for estimating the number of steps and total energy expenditure (TEE) (8,19,20). The SenseWear unit includes a dual-axial accelerometer to measure motion and sensors for skin temperature, heat flux, and galvanic skin response. Use of accelerometers consistently throughout the day and night can capture data on important health indicators such as TEE and sleep duration. Therefore, researchers developed a protocol to ask children to use the device for 7 consecutive days and nights.

**Table:** Specifications of Devices Used to Evaluate Feasibility of Using Wearable Devices to Measure Physical Activity of Children

Specification	Philips Actical	SenseWear by BodyMedia	Polar Active	Polar Heart Rate Monitor
**Cost per unit, including basic accessories**	$450	$120	$99	$135
**Other accessories required for use (cost)**	ActiReader device ($725); Actical software ($500)	Downloadable software (free)	FlowLink data transfer device ($50); Polargofit.com online system (free)	Polar Advantage interface and software (free)
**Number of axes**	1	2^a^	1	Not applicable
**Epoch length**	1, 2, 5, 15, 30, 60 sec	20 sec	30 sec	Not applicable
**Device placement**	Waist^b^	Upper arm	Wrist	Chest and wrist
**Data storage capacity**	12 days	28 days	21 days	134 h performance information
**Output measures (per 24 h period)**	TEE; active energy expenditure; duration and energy expended in 4 activity zones^c^	TEE; active energy expenditure; METS; total number of steps; physical activity levels and duration; on/off body time^d^>	TEE; sleep duration; total number of steps; duration of activity in 5 intensity zones^e^; time in MVPA	Average, minimum, and maximum heart rates; time below, within, and above the target zones; relaxation rate
**Moisture protection**	Not waterproof^f^	Not waterproof	Waterproof 30–50 min	Waterproof 30–50 min

Abbreviations: TEE, total energy expenditure; METS, metabolic equivalents; MVPA, moderate to vigorous physical activity.

a Most recent model available uses triaxial accelerometer.

b Comes with a waistband, wristband, and ankle band to allow various attachment sites.

c 4 activity zones include sedentary, light, moderate, and vigorous.

d Most recent model measures sleep duration and efficiency.

e 5 activity zones include very easy, easy, moderate, vigorous, vigorous +.

f Most recent model available is waterproof (1 m for 30 min).

Children aged 7 to 10 years were recruited via flyers and posters around Yolo County in schools and community centers and from faculty and staff at UC Davis. Children were provided information letters, and written informed consent was obtained from a parent or guardian. Children and their parents were scheduled for 5 appointments during 4 weeks at the WHNRC facility located on the UC Davis campus. During the first visit, children and parents were briefed on study procedures by research staff. Each child was asked to use a different device each week under parental supervision. It was specified that each device be worn at all times during the day and night, unless the child experienced discomfort, and removed only for water-based activities or as indicated (the Polar Active is waterproof and thus required no removal). Written instructions for each device were also provided, including how and when the device should be worn. Each parent received a daily log to record any device removals and the reason for doing so.

Each week, children accompanied by a parent visited the research center to return assigned devices, undergo an individual structured interview with trained research staff about that device (referred to hereafter as “device interview”), and receive a new device for use during the subsequent week. Device interviews consisted of 9 main questions (Box 1) that assessed the frequency of device removals, reasons for removal, enjoyment and comfort during use, and favorite and least favorite device characteristics. Each parent completed a similarly structured interview on their perceptions of their child’s experience with each device. All parent and child interviews were conducted in English, with the exception of 1 parent interview conducted in Spanish by the principal investigator, who is fluent in Spanish. On average, interviews lasted approximately 20 minutes.

**Box 1. Script for the Device Interview**

1. Did you wear the monitor every day for the past 7 days and nights? If not, for how many days or nights did you wear it?
2. How much of the time did you wear the monitor each day and night? Would you say all of the time? Most of the time? Some of the time? Why didn’t you wear it all of the time? [Further question, if necessary, to note any times the child took it off and why. Ask to describe any specific situations; eg, when I took a bath, when I got sweaty playing basketball.]
3. Did you at any time while you were wearing the monitor during the day or night, take it off? If so, why did you take it off?
4. On a scale of 0 to 5, how much did you like wearing the monitor? [5 = Liked extremely well to 0 = Did not like at all.] Why did you give this answer?
5. On a scale of 0 to 5, how comfortable was wearing the monitor for the time we asked you to wear it? [5 = Very comfortable to 0 = Not at all comfortable.] Why did you give this answer?
6. What did you like most about wearing the monitor?
7. What did you like least about wearing the monitor?
8. What else was hard about wearing the monitor when you wore it?
9. What do you think would make wearing the monitor better?

Upon completion of the 4-week study, each child (and parent) participated in an exit interview, during which they were asked to rank the devices by placing them in order from most to least preferred (Box 2). Research staff assigned a number to each child’s answer (on a scale of 1 to 4, with 1 = Least favorite and 4 = Favorite).

**Box 2. Script for the Exit Interview**

[Instructions to interviewer: Place the 4 devices in front of the child, from their left to right, in the order that they used them (eg, the device they wore first will be on their extreme left — your right — then the second device they wore will be on its right, followed by the third device, and lastly the fourth device that they wore will be on their right).]

1. Here are the 4 devices that you have worn, each for 7 days. Consider everything you felt when you wore these devices. Please rank the four devices in order from favorite (4) to least favorite (1). [Note subject’s rank order below:

______ Polar Active

______ Philips Actical

______ BodyMedia SenseWear

______ Polar heart rate monitor]

1. Why did you choose ______________ as your favorite?
2. Why did you choose ______________ as your least favorite?

Interview responses were examined and common responses counted for frequency of occurrence. Data analyses were conducted with SPSS Statistics version 19.0 (IBM Corporation, Armonk, New York) and Microsoft Excel version 12.3.1 (Microsoft Corporation, Redmond, Washington). Compliance was assessed by counting the number of reported removals (min/d) during day 2 through day 6 on the daily logs completed by parents. Removals due to water-related activities (bathing, swimming, etc.) were not counted because removal was required for nonwaterproof devices. Researchers measured each child’s perceived enjoyment and comfort in using each device by scoring items 4 (“On a scale of 0 to 5, how much did you like wearing the monitor?”) and 5 (“On a scale of 0 to 5, how comfortable was wearing the monitor for the time we asked you to wear it?”) of the device interview. Each child’s device preference was determined by their final ranking in the exit interview, and their ranking was compared with their parent’s ranking. Output data were examined for lost or missing data as a result of device malfunctioning or removal. Reactivity was examined for differences between the first (day 2) and last (day 6) complete day of activity measurement; we used a paired t test to detect differences in TEE and moderate to vigorous physical activity (MVPA) between the first and last day of device use.

## Results

Of the 25 children enrolled, 24 completed the 4-week study (11 boys, 13 girls); the average age was 8.9 years (standard deviation [SD], 1.3 y). One child was withdrawn from the study for personal issues unrelated to device use. All devices were returned undamaged. After 24 children completed the study, researchers determined that device and exit interviews were yielding consistent patterns of saturation.

Overall, children indicated the greatest preference for the wrist-worn Polar Active; it ranked an average of 3.9 of 4 (Figure), and 23 of 24 children ranked it as their favorite. Parent exit interviews showed a 90% agreement with child rankings. In exit interviews, the most common reasons for deeming this device the favorite were comfort (10 of 23 children) and the feedback feature (8 of 23 children). Other reasons included that it didn’t make noise, it was “cool,” and it showed the time. The least preferred device among children was the SenseWear, ranked the least favorite by 16 of 23 child participants. Reasons for the low ranking included that it was uncomfortable to wear (12 of 16 children), it was embarrassing to wear (5 of 16 children), and it made a lot of noise (4 of 16 children). The pattern of the children’s weekly ranking of each device for enjoyment and comfort corresponded to the pattern of the children’s final ranking during exit interviews.

**Figure:**
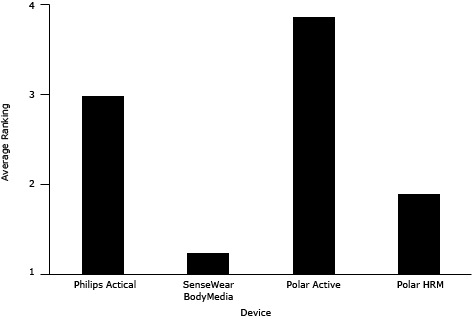
Average ranking for physical activity devices by children; scale is 1 to 4, with 4 being the favorite. Abbreviation: HRM, heart rate monitor.

Levels of compliance were highest among children wearing the Polar Active. During the 5 complete days of data collection, the Polar Active was used 98% of the time, the Actical was used 92% of the time, and the SenseWear was used 28% of the time. In device interviews, children indicated that the main reason for removing a device was discomfort, followed by forgetting to reattach the device after a removal (most often due to water-related activities) and for sports activities that prohibited use.

The protocol defined for this study allowed for the collection of up to 5 complete days of data from compliant children. High levels of compliance among children wearing the Polar Active meant that this device provided the most complete data of children’s daily physical activity. Thus, the patterns of the study group were determined by using data collected from this device. The distribution of participant time in moderate to vigorous physical activity (MVPA) showed that, on average, 11 of the 24 children (46%) met or exceeded the recommended 60 minutes per day. Daily sleep time totaled an average 584.9 (SD, 198.5) minutes, or 9.7 (SD, 3.3) hours; total energy expenditure was 1,592.5 (SD, 265.4) kilocalories per day; and number of steps per day was 19,005.2 (SD, 6,894.1). Reactivity was assessed only among children wearing the Polar Active because of the excessive removal of other devices and the associated loss of data. We found no differences in TEE or MVPA between the first and last day of device use.

## Discussion

This study was conducted to examine the feasibility of accelerometers for use in research studies and programs involving pre-adolescent children. Of the devices assessed in this study, the wrist-worn device (Polar Active) had the highest levels of acceptability and compliance among children tested. Researchers perceived several benefits of using the Polar Active in this study population. Compared with other attachment sites (eg, hip, upper arm), the wrist appeared to be the most familiar site for children. This device required little instruction to the user, which researchers found helpful as they worked with the children. This device is waterproof, which researchers deemed a valuable characteristic, enabling the capture of data during water-based activities, lowering the occurrence of device removal, and decreasing the risk of damage to the device (16).

The Polar Active was not originally developed for research objectives but rather as tool for use in school-based physical education (21). One potential drawback of using the Polar Active in a research context is the real-time visual-feedback features on the watch face. These feedback features include an activity bar that displays the amount of time in the moderate to vigorous intensity zones and an animated figure that indicates activity intensity. In research interventions, blinding is important to prevent bias (22), and feedback may engage users in their current activity levels and shift their behavior. The lack of reactivity among Polar Active users in this study suggests these children did not change their behavior as a result of wearing this device. However, this study population was small, and these results may lack generalizability. Further research is needed to determine the effect of personalized real-time feedback in health monitoring and behavior change (23,24). Researchers had additional challenges in using the Polar Active because of the online system (polargofit.com) required to format the devices. The system is designed to format a device to be used by a single student over a long duration (such as a school year). However, in a research context, a device may be required for use by multiple users over shorter durations. It is possible to format the Polar Active for multiple users and shorter durations, but doing so requires time-consuming manipulations of the online system (25). Furthermore, although user activity data were provided by the system in Microsoft Excel format, the data were downloadable only in preformatted worksheets. The worksheets prohibit data extraction, thus requiring manual data entry, which is time consuming and carries a higher risk of error. In this aspect, the Actical and SenseWear may be better suited for research studies with groups or populations.

The SenseWear had the lowest levels of compliance and acceptability among this study population. These low levels were at least partially attributed to the device not being sized appropriately for pre-adolescent children (despite use of a size small armband). Children and their parents reported frequent difficulty in maintaining firm placement of the device on the upper arm, which was a major cause of discomfort. Furthermore, this device requires skin contact for activation, upon which it beeps and vibrates. Intermittent deactivation and activation was experienced by many children because of the loose fit and was reported as bothersome and disruptive while the children were in school. The intermittent deactivation also resulted in frequent data loss. These findings highlight the importance of proper fit when using activity-monitoring devices on children.

Price can play an important role in selecting a device to assess physical activity in a study population. Cost analysis showed a wide price range among devices with similar capabilities (Table). Financial considerations can restrict the quantity of units that can be purchased and consequently restrict the size and scope of studies. Thus, in research studies with medium to large sample sizes, price can be a determining factor in device selection.

Although there are many benefits to using accelerometers to measure physical activity compared with other methods, these devices are not without limitations. Vertical acceleration of the body can be measured accurately with unidirectional accelerometers, but they do not accurately reflect external work performed in such activities as pushing or lifting objects, stair climbing, cycling, rowing, or resistance training (12,26,27). Many accelerometers, especially older models, are not waterproof and therefore cannot measure water-based activities. Another limitation of using accelerometers is the variability among devices in detection and output. Accelerometer output, referred to as “counts,” represents the estimated intensity of measured activities during each time period, allowing for the calculation of time spent at differing intensities of activity. However, intensity cut points vary among instruments. Evidence is unclear as to whether choice of cut points matters when measuring associations of activity with health outcomes. Regardless, threshold agreement among devices would allow for meaningful evaluation of a population’s physical activity relative to national recommendations (8), not to mention better comparability between studies using different devices. Another limitation of accelerometers is that they can be impractical to wear. As experienced by at least 1 child in this study, individuals may not be able to wear devices during competitive or high-contact sports. This limitation has clear implications for measuring moderate to vigorous physical activity (28–30).

Many accelerometers do not provide information on the type or context of physical activity, with the exception of devices that have multiple sensors, such as the SenseWear. Other activity-monitoring devices on the market or in development include sensors or algorithms for providing information on sleep duration, patterns, and efficiency; heart rate; stress; speed; and distance. Advances in technology are expected to soon lead to less expensive, smaller devices. Some of the devices in this study have since released newer models that reflect some of these changes.

This study highlights the importance of acceptability of wearable activity monitors among children. Devices that are comfortable to wear, fit properly, have engaging features, and are waterproof increase feasibility and are associated with higher levels of compliance. Accelerometers worn on the wrist may be more acceptable among children than devices worn on the upper arm or hip. User feedback is not a desirable feature from the researcher point of view because of its potential to shift behavior, but it does increase engagement of the child user, contributing to improved compliance; the effect of feedback warrants further study. 
